# MRI-based Alzheimer’s disease-resemblance atrophy index in the detection of preclinical and prodromal Alzheimer’s disease

**DOI:** 10.18632/aging.203082

**Published:** 2021-05-25

**Authors:** Wanting Liu, Lisa Wing Chi Au, Jill Abrigo, Yishan Luo, Adrian Wong, Bonnie Yin Ka Lam, Xiang Fan, Pauline Wing Lam Kwan, Hon Wing Ma, Anthea Yee Tung Ng, Sirong Chen, Eric Yim Lung Leung, Chi Lai Ho, Simon Ho Man Wong, Winnie CW Chu, Ho Ko, Alexander Yuk Lun Lau, Lin Shi, Vincent Chung Tong Mok, Alzheimer’s Disease Neuroimaging Initiative

**Affiliations:** 1 Division of Neurology, Department of Medicine and Therapeutics, Therese Pei Fong Chow Research Centre for Prevention of Dementia, The Chinese University of Hong Kong, Hong Kong SAR, China; 2 Gerald Choa Neuroscience Centre, Lui Che Woo Institute of Innovative Medicine, The Chinese University of Hong Kong, Hong Kong SAR, China; 3 Department of Imaging and Interventional Radiology, The Chinese University of Hong Kong, Prince of Wales Hospital, Hong Kong SAR, China; 4 BrainNow Research Institute, Hong Kong Science and Technology Park, Hong Kong SAR, China; 5 Department of Nuclear Medicine and PET, Hong Kong Sanatorium and Hospital, Hong Kong SAR, China; 6 Medhealth Diagnostic MRI Centre, Hong Kong SAR, China; 7 Li Ka Shing Institute of Health Sciences, School of Biomedical Sciences, Faculty of Medicine, The Chinese University of Hong Kong, Hong Kong SAR, China

**Keywords:** preclinical Alzheimer’s disease, prodromal Alzheimer's disease, volumetric segmentation tool, MRI, amyloid and tau PET

## Abstract

Alzheimer’s Disease-resemblance atrophy index (AD-RAI) is an MRI-based machine learning derived biomarker that was developed to reflect the characteristic brain atrophy associated with AD. Recent study showed that AD-RAI (≥0.5) had the best performance in predicting conversion from mild cognitive impairment (MCI) to dementia and from cognitively unimpaired (CU) to MCI. We aimed to validate the performance of AD-RAI in detecting preclinical and prodromal AD. We recruited 128 subjects (MCI=50, CU=78) from two cohorts: CU-SEEDS and ADNI. Amyloid (A+) and tau (T+) status were confirmed by PET (^11^C-PIB, ^18^F-T807) or CSF analysis. We investigated the performance of AD-RAI in detecting preclinical and prodromal AD (i.e. A+T+) among MCI and CU subjects and compared its performance with that of hippocampal measures. AD-RAI achieved the best metrics among all subjects (sensitivity 0.74, specificity 0.91, accuracy 85.94%) and among MCI subjects (sensitivity 0.92, specificity 0.81, accuracy 86.00%) in detecting A+T+ subjects over other measures. Among CU subjects, AD-RAI yielded the best specificity (0.95) and accuracy (85.90%) over other measures, while hippocampal volume achieved a higher sensitivity (0.73) than AD-RAI (0.47) in detecting preclinical AD. These results showed the potential of AD-RAI in the detection of early AD, in particular at the prodromal stage.

## INTRODUCTION

Detection of subjects at risk of developing dementia associated with Alzheimer’s disease (AD) and intervention at the early stage provides the greatest opportunity in reducing the increasing dementia burden associated with AD, which is the commonest cause for dementia among the older population. The latest 2018 National Institute on Aging and Alzheimer’s Association (NIA-AA) research framework defined AD biologically by the presence of 2 core pathologic molecular biomarkers, amyloid-β (A+) and neurofibrillary tau (T+), rather than by the presence of cognitive impairment [1]. With this definition, subjects harboring A+T+ may exhibit a continuum of severity of cognitive impairment, ranging from cognitively unimpaired (CU) (i.e. preclinical AD), to mild cognitive impairment (MCI) (i.e. prodromal AD), to dementia (i.e. AD with dementia). The evolution from preclinical to prodromal AD, or from prodromal AD to AD with dementia may take several years and this slow transition provides an excellent window to implement strategies that may prevent conversion to dementia.

This shift in paradigm (i.e. from reliance on clinical symptoms to molecular biomarkers, from focusing on dementia to pre-dementia stage) makes having an accurate *in-vivo* method in detecting AD biomarkers to be of great importance. At present, accurate *in-vivo* detection of beta-amyloid and neurofibrillary tau is feasible with positron emission tomography (PET) and cerebrospinal fluid (CSF) analysis. Studies comparing antemortem amyloid and tau PET and CSF analysis of beta-amyloid_1-42_ (Aβ_1–42_) and phosphorylated tau (p-tau) showed excellent correlation with post-mortem amyloid and tau burden [2–4]. Both PET and/or CSF are currently considered as the gold standard *in-vivo* diagnostic tests for preclinical and prodromal AD.

Apart from beta-amyloid and neurofibrillary tau, the 2018 NIA-AA research framework also considers neurodegeneration (N) as another biomarker for AD [1]. However, neurodegeneration is considered a downward and relatively more advanced event in the biological cascade of AD progression and is also non-specific, as many other brain diseases may also cause neurodegeneration. Neurodegeneration in AD is currently captured *in-vivo* by Fluorodeoxyglucose (FDG) PET hypometabolism, CSF total-tau, and atrophy on magnetic resonance imaging (MRI). Despite being considered as an advanced event in the biological cascade of AD, previous studies suggested that subtle yet characteristic pattern of neurodegeneration could still be detected by FDG PET, CSF total-tau, or MRI at the preclinical or prodromal stage of AD [5–8]. Moreover, subjects with A+T+(N)+ are at higher risk of future cognitive decline than those with A+T+(N)- [9, 10]. Hence, detection of characteristic pattern of neurodegeneration may have a role in the detection or prognostication for preclinical and prodromal AD.

Among the 3 conventional modalities in capturing neurodegeneration in AD, only MRI is non-invasive and is relatively more accessible than PET and CSF analysis. Structural MRI can capture the unique pattern of brain atrophy associated with AD, which is more prominent in the medial temporal lobe (e.g. hippocampus) initially, and then spread throughout the entire temporal lobe, parietal lobe, and frontal lobe [5, 8, 11]. Medial temporal lobe atrophy (MTA) or hippocampal volume (HV) as determined by MRI is the commonest imaging biomarker used for the diagnosis of AD with dementia or as a prognostic biomarker predicting conversion from MCI to AD with dementia [12, 13]. With the advancement of MRI-based automated brain segmentation tools, global and regional brain volumes (e.g. HV) can now be quantified accurately, reliably, easily, and quickly. In addition, several studies attempted to combine multi-region brain atrophy features on MRI in the form of a single severity index as derived from machine learning method and investigated its accuracy in predicting risk of conversion from MCI to dementia or from CU to MCI at an individual level [14–19]. We recently showed that a MRI-based machine learning derived AD-resemblance atrophy index (AD-RAI) had the best prognostic performance over other regional volumetric measures in predicting conversion from MCI to dementia and from CU to MCI using subjects from the AD Neuroimaging Initiatives-2 (ADNI-2) [19]. This index indicates the similarity in atrophy pattern between the subject’s brain and those with AD with dementia. It ranges from 0 to 1.0 and value closer to 1 implies greater similarity. The optimal AD-RAI cutoff of differentiating converters from non-converters derived from subjects recruited from ADNI was ≥ 0.5 [19].

In this study, we aimed to validate the performance of AD-RAI at the cutoff of ≥ 0.5 obtained from our recent derivation study [19] in the detection of preclinical and prodromal AD among MCI and CU subjects recruited from our prospective cohort and the ADNI cohort (excluding ADNI-2), and to compare its performance with that of traditional MRI-based measures, namely visual MTA rating and quantitative hippocampal measures. We hypothesized that AD-RAI is able to reflect the characteristic pattern of brain atrophy that is associated with A+T+ at the prodromal or preclinical stage of AD.

## RESULTS

We recruited 138 patients altogether. Apart from 128 subjects with MCI (n=50) and CU (n=78) (Table 1A), we also recruited 10 subjects with AD-like dementia for the validation of our PET protocols. The demographic and clinical characteristics of MCI and CU subjects in each cohort are shown in Table 1B. Intra-rater reliability for visual MTA rating showed a weighted Kappa of 0.74. The test/re-test precision of AccuBrain^®^ in generating repeated measures was perfect (i.e. 100%) for AD-RAI, HV, and HF.

**Table 1A:** Demographic and clinical characteristics of subjects.

	**All subjects (n=128)**	**MCI (n=50)**	**CU (n=78)**	**P-value**
Age (years), mean (SD)	68.42 ± 6.21	69.80 ± 6.26	67.54 ± 6.05	0.044*
Male (n [%])	64 (50.0)	28 (56.0)	36 (46.2)	0.281
Education (years), mean (SD)	12.48 ± 5.35	12.3 ± 5.68	12.61 ± 5.16	0.755
A+T+ (n [%])	39 (30.5)	24 (48.0)	15 (19.2)	0.001*
A+T- (n [%])	9 (7.0)	4 (8.0)	5 (6.4)	0.734
CDR, mean (SD)	0.20 ± 0.24	0.5	0	NA**
AD-RAI, mean (SD)	0.29 ± 0.35	0.52 ± 0.38	0.15 ± 0.23	< 0.001*
HV (mL), mean (SD)	6.69 ± 0.88	6.32 ± 0.81	6.94 ± 0.84	< 0.001*
HF (%), mean (SD)	0.46 ± 0.05	0.43 ± 0.05	0.47 ± 0.05	< 0.001*

MCI=mild cognitive impairment; CU=cognitively unimpaired; SD=standard deviation; A+T+=subjects harboring beta-amyloid and tau; A+T-=subjects harboring beta-amyloid only; CDR= clinical dementia rating scale; AD-RAI= Alzheimer’s disease resemblance atrophy index; HV=hippocampal volume; HF=hippocampal fraction. The p-values represent the group difference in each variable between MCI subgroup and CU subgroup derived from independent-samples t-test. *represents significant difference at p < 0.05. **T-test cannot be computed because the standard deviations of both groups are 0.

**Table 1B:** Demographic and clinical characteristics of subjects in CU-SEEDS and ADNI cohorts.

	**CU-SEEDS**				**ADNI**			
	**All subjects** **(n=64)**	**MCI** **(n=25)**	**CU** **(n=39)**	**P-value**	**All subjects** **(n=64)**	**MCI** **(n=25)**	**CU** **(n=39)**	**P-value**
Age (years), mean (SD)	66.75 ± 6.99	69.80 ± 6.49	64.80 ± 6.65	0.004*	70.10 ± 4.81	69.80 ± 6.15	70.29 ± 3.81	0.723
Male (n [%])	27 (42.2)	11 (44.0)	16 (41.0)	0.818	37 (57.8)	17 (68.0)	20 (51.3)	0.187
Education (years), mean (SD)	9.19 ± 4.60	8.28 ± 4.95	9.81 ± 4.30	0.201	15.67 ± 3.92	16.32 ± 2.77	15.26 ± 4.49	0.293
A+T+ (n [%])	15 (23.4)	11 (44.0)	4 (10.3)	0.005*	24 (37.5)	13 (52.0)	11 (28.21)	0.238
A+T- (n [%])	3 (4.7)	2 (8.0)	1 (2.6)	0.379	6 (9.4)	2 (8.0)	4 (10.3)	0.767
CDR, mean (SD)	0.20 ± 0.25	0.5	0	NA	0.20 ± 0.25	0.5	0	NA
HK-MoCA, mean (SD)	24.49 ± 4.57	21.08 ± 4.51	26.70 ± 3.02	< 0.001*	NA	NA	NA	NA
MMSE, mean (SD)	NA	NA	NA	NA	28.31 ± 1.71	27.28 ± 1.90	28.97 ± 1.18	< 0.001*
^11^C-PIB global retention, mean (SD)	1.36 ± 0.18	1.43 ± 0.20	1.31 ± 0.15	0.010*	NA	NA	NA	NA
T807 global SUVR, mean (SD)	1.07 ± 0.10	1.11 ± 0.13	1.05 ± 0.07	0.045*	NA	NA	NA	NA
CSF Aβ_1–42_ (pg/ml), mean (SD)	NA	NA	NA	NA	193.17 ± 56.81	176.88 ± 55.63	203.62 ± 55.76	0.066
CSF p-tau (pg/ml), mean (SD)	NA	NA	NA	NA	30.87 ± 18.44	36.21 ± 19.47	27.44 ± 17.13	0.063
AD-RAI, mean (SD)	0.25 ± 0.34	0.47 ± 0.39	0.11 ± 0.19	< 0.001*	0.34 ± 0.36	0.57 ± 0.37	0.19 ± 0.26	< 0.001*
HV (mL), mean (SD)	6.91 ± 0.89	6.35 ± 0.80	7.27 ± 0.75	< 0.001*	6.48 ± 0.82	6.29 ± 0.84	6.60 ± 0.79	0.136
HF (%), mean (SD)	0.48 ± 0.05	0.45 ± 0.03	0.50 ± 0.04	< 0.001*	0.43 ± 0.05	0.42 ± 0.06	0.44 ± 0.04	0.046*

CU-SEEDS= The Chinese University of Hong Kong - Screening for Early Alzheimer’s Disease; ADNI= Alzheimer’s Disease Neuroimaging Initiatives; MCI=mild cognitive impairment; CU=cognitively unimpaired; SD=standard deviation; A+T+=subjects harboring beta-amyloid and tau; A+T-=subjects harboring beta-amyloid only; CDR= clinical dementia rating scale; HK-MoCA=Hong Kong version of Montreal Cognitive Assessment; MMSE=Mini Mental State Examination; 11C-PIB=11C-Pittsburgh compound B; SUVR= standardized uptake value ratio; CSF= cerebrospinal fluid; Aβ1–42=beta-amyloid1-42; p-tau=phosphorylated tau; AD-RAI= Alzheimer’s disease resemblance atrophy index; HV=hippocampal volume; HF=hippocampal fraction. The p-values represent the group difference in each variable between MCI subgroup and CU subgroup derived from independent-samples t-test. *represents significant difference at p < 0.05. **T-test cannot be computed because the standard deviations of both groups are 0.

Number (percentage) of subjects who were A+T+ among dementia, MCI, and CU were 10 (100%), 24 (48%), and 15 (19.2%), respectively. The findings that all the 10 dementia subjects were both A+ and T+ (i.e. 100%) lent support to the sensitivity and validity of the PET protocols of CU-SEEDS. Performance of AD-RAI and other imaging measures in the detection of A+T+ among the 10 dementia subjects can be found in Supplementary Table 2. In brief, AD-RAI (≥ 0.5) yielded the best sensitivity (i.e. 0.90) in detecting A+T+ among dementia subjects when compared with HV (0.80), HF (0.50), and visual MTA (0.80).

Among all subjects (i.e. MCI and CU subjects) (Table 2A), AD-RAI (≥ 0.5) yielded the best sensitivity (0.74) and accuracy (85.94%) over other measures, as well as a high specificity of 0.91 in detecting AD (A+T+) subjects. HV (≤ 6.44mL) yielded a fair sensitivity of 0.69, with a specificity of 0.75 and an accuracy of 73.44%. HF (≤ 0.42%) had the highest specificity of 0.92, yet with a fair sensitivity of 0.51. Sensitivity, specificity, and accuracy of MTA (≥ 1) were 0.51, 0.88, and 76.56%, respectively.

**Table 2A:** Performance metrics of AD-RAI, HV, HF and MTA among MCI and CU subjects in the detection of A+T+ (n=128).

**Measures**	**Sensitivity (95% CI)**	**Specificity (95% CI)**	**Positive predictive value**	**Negative predictive value**	**Accuracy**
AD-RAI (≥0.5)	0.74 (0.58-0.86)	0.91 (0.83-0.96)	78.38%	89.01%	85.94%
HV (≤ 6.44mL)	0.69 (0.52-0.82)	0.75 (0.65-0.84)	55.10%	84.81%	73.44%
HF (≤ 0.42%)	0.51 (0.35-0.67)	0.92 (0.84-0.97)	74.07%	81.19%	79.69%
MTA (≥ 1)	0.51 (0.35-0.67)	0.88 (0.79-0.93)	64.52%	80.41%	76.56%

MCI=mild cognitive impairment; CU=cognitively unimpaired; AD-RAI=Alzheimer’s disease resemblance atrophy index; HV=hippocampal volume; HF=hippocampal fraction; MTA=medial temporal lobe atrophy; CI=confidence interval.

Among MCI subjects (Table 2B), AD-RAI (≥ 0.5) yielded the best metrics over other measures, with an excellent sensitivity of 0.92, a good specificity of 0.81, and a good overall accuracy of 86.00% in detecting prodromal AD. HV (≤ 6.07mL) yielded a high specificity of 0.88, yet with a lower sensitivity (0.71) and accuracy (80.00%). HF (≤ 0.41%) yielded a fair sensitivity of 0.58, a high specificity of 0.88, and an accuracy of 74.00%. Sensitivity, specificity, and accuracy of MTA (≥ 1) were 0.67, 0.81, and 74.00%, respectively.

**Table 2B:** Performance metrics of AD-RAI, HV, HF and MTA among MCI subjects in the detection of A+T+ (n=50).

**Measures**	**Sensitivity (95% CI)**	**Specificity (95% CI)**	**Positive predictive value**	**Negative predictive value**	**Accuracy**
AD-RAI (≥ 0.5)	0.92 (0.72-0.99)	0.81 (0.60-0.93)	81.48%	91.30%	86.00%
HV (≤ 6.07mL)	0.71 (0.49-0.87)	0.88 (0.69-0.97)	85.00%	76.67%	80.00%
HF (≤ 0.41%)	0.58 (0.37-0.77)	0.88 (0.69-0.97)	82.35%	69.70%	74.00%
MTA (≥ 1)	0.67 (0.45-0.84)	0.81 (0.60-0.93)	76.19%	72.41%	74.00%

AD-RAI=Alzheimer’s disease resemblance atrophy index; HV=hippocampal volume; HF=hippocampal fraction; MTA=medial temporal lobe atrophy; MCI=mild cognitive impairment; CI=confidence interval.

Among CU subjects (Table 2C), AD-RAI yielded the highest specificity (0.95) and accuracy (85.90%), yet with a low sensitivity of 0.47 in detecting preclinical AD. HV (≤ 6.64mL) yielded a higher sensitivity (0.73) than AD-RAI, along with a fair specificity (0.70) and accuracy (70.51%). HF (≤ 0.44%) yielded a low sensitivity of 0.47, a high specificity of 0.87 and an accuracy of 91.79%. Sensitivity, specificity, and accuracy of MTA (≥ 1) were 0.27, 0.90, and 78.21%, respectively.

**Table 2C:** Performance metrics of AD-RAI, HV, HF and MTA among CU subjects in the detection of A+T+ (n=78).

**Measures**	**Sensitivity (95% CI)**	**Specificity (95% CI)**	**Positive predictive value**	**Negative predictive value**	**Accuracy**
AD-RAI (≥ 0.5)	0.47 (0.22-0.73)	0.95 (0.86-0.99)	70.00%	88.24%	85.90%
HV (≤ 6.64mL)	0.73 (0.45-0.91)	0.70 (0.57-0.80)	36.67%	91.67%	70.51%
HF (≤ 0.44%)	0.47 (0.22-0.73)	0.87 (0.76-0.94)	46.67%	87.30%	71.79%
MTA (≥ 1)	0.27 (0.09-0.55)	0.90 (0.80-0.96)	40.00%	83.82%	78.21%

AD-RAI=Alzheimer’s disease resemblance atrophy index; HV=hippocampal volume; HF=hippocampal fraction; MTA=medial temporal lobe atrophy; CU=cognitively unimpaired; CI=confidence interval.

We performed separate analysis on the performance of various imaging measures in detecting subjects harboring A+T+ in respective cohorts. Results of these analysis are shown in Table 3A–3C. In general, the performance metrics of AD-RAI were similar between these two cohorts.

**Table 3A:** Performance metrics of AD-RAI, HV, HF, and MTA among MCI and CU subjects in the detection of A+T+ in CU-SEEDS (n=64) and ADNI cohorts (n=64).

**Measures**	**Sensitivity (95% CI)**		**Specificity (95% CI)**		**Positive predictive value**		**Negative predictive value**		**Accuracy**	
	**CU-SEEDS**	**ADNI**	**CU-SEEDS**	**ADNI**	**CU-SEEDS**	**ADNI**	**CU-SEEDS**	**ADNI**	**CU-SEEDS**	**ADNI**
**AD-RAI** **(≥ 0.5)**	0.73 (0.45-0.91)	0.75 (0.53-0.89)	0.92 (0.80-0.97)	0.90 (0.75-0.97)	73.33%	81.82%	91.84%	85.71%	87.50%	84.38%
**HV** **(≤ 6.44mL)**	0.67 (0.39-0.87)	0.71 (0.49-0.87)	0.86 (0.72-0.94)	0.63 (0.46-0.77)	58.82%	53.13%	89.36%	78.13%	81.25%	65.63%
**HF** **(≤ 0.42%)**	0.27 (0.09-0.55)	0.67 (0.45-0.84)	1.00 (0.91-1.00)	0.83 (0.67-0.92)	100.00%	69.57%	81.67%	80.49%	82.81%	76.56%
**MTA** **(≥ 1)**	0.53 (0.27-0.78)	0.50 (0.30-0.70)	0.92 (0.80-0.97)	0.83 (0.67-0.92)	66.67%	63.16%	86.54%	73.33%	82.81%	70.31%

AD-RAI=Alzheimer’s disease resemblance atrophy index; HV=hippocampal volume; HF=hippocampal fraction; MTA=medial temporal lobe atrophy; MCI=mild cognitive impairment; CU=cognitively unimpaired; CU-SEEDS= The Chinese University of Hong Kong - Screening for Early Alzheimer’s Disease; ADNI= Alzheimer’s Disease Neuroimaging Initiatives; CI=confidence interval.

**Table 3B:** Performance metrics of AD-RAI, HV, HF, and MTA among MCI subjects in the detection of A+T+ in CU-SEEDS (n=25) and ADNI cohorts (n=25).

**Measures**	**Sensitivity (95% CI)**		**Specificity (95% CI)**		**Positive predictive value**		**Negative predictive value**		**Accuracy**	
	**CU-SEEDS**	**ADNI**	**CU-SEEDS**	**ADNI**	**CU-SEEDS**	**ADNI**	**CU-SEEDS**	**ADNI**	**CU-SEEDS**	**ADNI**
**AD-RAI** **(≥ 0.5)**	0.91 (0.57-1.00)	0.92 (0.62-1.00)	0.79 (0.49-0.94)	0.83 (0.51-0.97)	76.92%	85.71%	91.67%	90.91%	84.00%	88.00%
**HV** **(≤ 6.07mL)**	0.64 (0.32-0.88)	0.77 (0.46-0.94)	1.00 (0.73-1.00)	0.75 (0.43-0.93)	100.00%	76.92%	78.78%	75.00%	84.00%	76.00%
**HF** **(≤ 0.41%)**	0.27 (0.07-0.61)	0.85 (0.54-0.97)	1.00 (0.73-1.00)	0.75 (0.43-0.93)	100.00%	78.57%	63.63%	81.82%	68.00%	80.00%
**MTA** **(≥ 1)**	0.64 (0.32-0.88)	0.69 (0.39-0.90)	0.79 (0.49-0.94)	0.83 (0.51-0.97)	70.00%	81.82%	73.33%	71.43%	72.00%	76.00%

AD-RAI=Alzheimer’s disease resemblance atrophy index; HV=hippocampal volume; HF=hippocampal fraction; MTA=medial temporal lobe atrophy; MCI=mild cognitive impairment; CU-SEEDS= The Chinese University of Hong Kong - Screening for Early Alzheimer’s Disease; ADNI= Alzheimer’s Disease Neuroimaging Initiatives; CI=confidence interval.

**Table 3C:** Performance metrics of AD-RAI, HV, HF, and MTA among CU subjects in the detection of A+T+ in CU-SEEDS (n=39) and ADNI cohorts (n=39).

**Measures**	**Sensitivity (95% CI)**		**Specificity (95% CI)**		**Positive predictive value**		**Negative predictive value**		**Accuracy**	
	**CU-SEEDS**	**ADNI**	**CU-SEEDS**	**ADNI**	**CU-SEEDS**	**ADNI**	**CU-SEEDS**	**ADNI**	**CU-SEEDS**	**ADNI**
**AD-RAI** **(≥ 0.5)**	0.25 (0.01-0.78)	0.55 (0.25-0.82)	0.97 (0.83-1.00)	0.93 (0.75-0.99)	50.00%	75.00%	91.89%	83.87%	89.74%	82.05%
**HV** **(≤ 6.64mL)**	0.75 (0.22-0.99)	0.73 (0.39-0.93)	0.89 (0.72-0.96)	0.46 (0.28-0.66)	42.86%	34.78%	96.88%	81.25%	87.18%	53.85%
**HF** **(≤ 0.44%)**	0.25 (0.01-0.78)	0.55 (0.25-0.82)	0.97 (0.83-1.00)	0.75 (0.55-0.89)	50.00%	46.15%	91.89%	80.77%	89.74%	69.23%
**MTA** **(≥ 1)**	0.25 (0.01-0.78)	0.27 (0.07-0.61)	0.97 (0.83-1.00)	0.82 (0.62-0.93)	50.00%	37.50%	91.89%	74.19%	89.74%	66.67%

AD-RAI=Alzheimer’s disease resemblance atrophy index; HV=hippocampal volume; HF=hippocampal fraction; MTA=medial temporal lobe atrophy; CU=cognitively unimpaired; CU-SEEDS= The Chinese University of Hong Kong - Screening for Early Alzheimer’s Disease; ADNI= Alzheimer’s Disease Neuroimaging Initiatives; CI=confidence interval.

The metrics of various imaging measures in detecting subjects harboring A+ with or without T (i.e. A+T+ and A+T-) can be found in Supplementary Table 3A–3C. Overall, almost all imaging measures had lower sensitivity and accuracy in detecting A+ with or without T when compared to that in detecting A+T+.

## DISCUSSION

In the present validation study, using the cutoff derived previously from the ADNI-2 database (i.e. ≥ 0.5) [19], AD-RAI achieved the best performance (sensitivity 0.74, specificity 0.91, accuracy 85.94%) in identifying AD subjects (i.e. A+T+) when compared with visual (MTA) and quantitative hippocampal measures (i.e. HV, HF) among subjects with mild or no cognitive impairment. Among MCI subjects, AD-RAI also yielded the best metrics when compared with other measures in detecting prodromal AD. Among CU subjects, AD-RAI yielded the best specificity (0.95) and accuracy (85.90%) over other measures, while HV achieved a higher sensitivity (0.73) than AD-RAI (sensitivity 0.47) in detecting preclinical AD. Overall, this study validated the performance of AD-RAI at the pre-specified cutoff of ≥ 0.5 in detecting early AD and supported the hypothesis that the pattern and severity of brain atrophy or neurodegeneration as reflected by MRI-based AD-RAI can aid the detection of early AD, in particular at the prodromal stage. To date, this is the first *in vivo* study exploring the performance of MRI-based machine learning method in detecting preclinical and prodromal AD as defined by the 2018 NIA-AA research framework, i.e. by the presence A+ and T+. Previous *in vivo* studies mainly investigated the ability of MRI-based machine learning methods in differentiating between converters and non-converters without knowledge of subjects’ amyloid and tau status [14–18].

**Figure 1:**
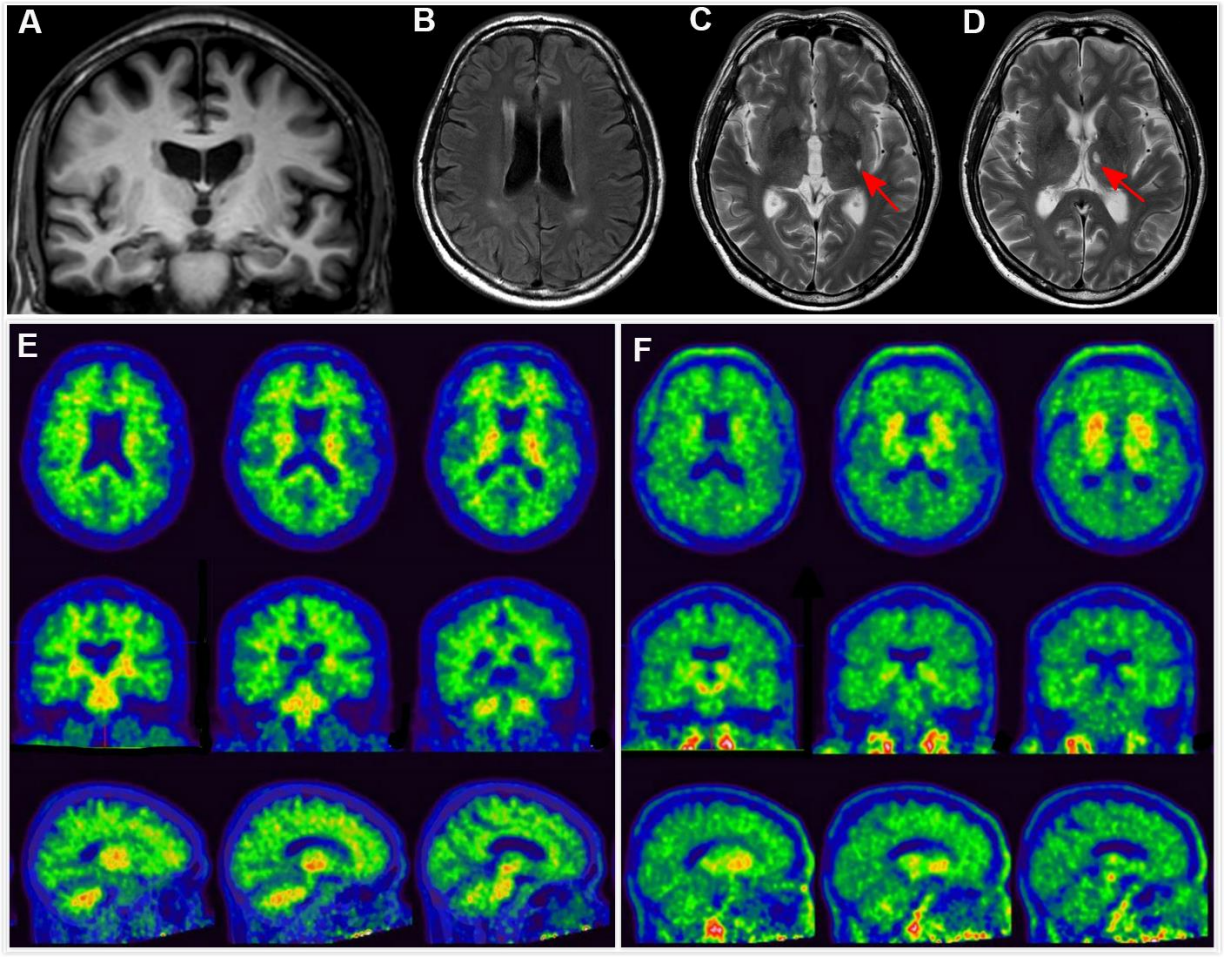
**Clinical utility of AD-RAI in MCI subjects.** A 68-year-old man with 11 years of education had complaints of memory decline for over 3 years. Z-score in Trial 4 of HKLLT was -1.94 SD (≤ -1 SD, i.e. MCI). Visual MTA rating score on MRI was 1 (≥ 1), (**A**) which was suggestive of AD. However, HV measures yielded conflicting results, with HF of 0.47% (> 0.41%) and raw HV of 7.38ml (> 6.07mL) suggestive of non-AD. FLAIR and T2-weighted sequences showed periventricular white matter hyperintensity and two subcortical lacunes (red arrows) (**B**–**D**). AD-RAI was only 0.11 (< 0.5) also suggestive of non-AD. Subsequent PIB PET (**E**) and T807 PET (**F**) showed negative results (i.e. A-T-), supporting the finding of AD-RAI. The MCI syndrome and mild MTA might be associated with cerebral SVD (i.e. vascular MCI associated with SVD).

Although there is still no definitive pharmacological treatment approved for preventing subjects with prodromal AD from progressing to AD with dementia, emerging studies have shown promising results of various strategies in slowing cognitive decline at an early or prodromal stage [20, 21]. Moreover, making a diagnosis of prodromal AD among subjects with MCI is also important for the sake of providing a correct diagnosis of the MCI syndrome, for prognostication, as well as for recruiting prodromal AD subjects into preventive clinical trials. Recent trials for AD have shifted to targeting subjects from the dementia stage to the prodromal or even preclinical stage [22]. Although PET or CSF analyses are now available to detect A+T+ at the early stage and have been used to recruit prodromal or preclinical AD subjects into clinical trials, availability of an easier method in detecting A+T+ subjects will help to reduce the cost of conducting clinical trials. Among MCI subjects, AD-RAI (≥ 0.5) achieved a high NPV of 91.30%, hence a “negative” AD-RAI will first help to rule out subjects without AD. For subjects with a “positive” AD-RAI, further investigations (i.e. PET or CSF analyses) can be arranged to confirm the diagnosis of prodromal AD. Moreover, using MRI as an initial investigation in MCI is also useful in ruling out other common brain lesions, e.g. cerebral small vessel disease (Figure 1) or other rare yet potential reversible causes, e.g. normal pressure hydrocephalus, brain tumor.

Noteworthy is that among our MCI subjects, only less than half of them (48%) had A+T+. This frequency is very similar to a meta-analysis showing that prevalence of amyloid positivity in MCI subjects at age of around 70-year-old (i.e. age similar to our MCI subjects) was around 50% [23]. Overall, the prevalence of amyloid positivity ranges from about 30% at age 50-year-old to 60% at age 80-year-old in MCI subjects [23]. This highlights the need of having additional tool to aid the detection of A+T+ among subjects presenting with MCI syndrome.

Among CU subjects, AD-RAI obtained the best specificity (0.95) and accuracy (85.90%) in the detection of preclinical AD, although its sensitivity was low (0.47). Given the very high specificity of 0.95, CU subjects who have a “positive” AD-RAI are very likely to have preclinical AD. In comparison, HV achieved a higher sensitivity of 0.73 than AD-RAI in the detection of preclinical AD. A recent study also showed that HV measure had acceptable accuracy in predicting conversion from normal to MCI [24]. Note that in our recent study [19], although AD-RAI achieved the best specificity (0.98) and accuracy (79.45%) over other measures, its sensitivity was also lower (0.39) than that of HV (0.70) (see Supplementary Material). Overall, the higher sensitivity of HV over AD-RAI is consistent with our current understanding on the temporal evolution of brain atrophy in AD, which is most apparent mainly in the hippocampus at the very early stage (e.g. preclinical stage), followed by spreading to other regions as disease progresses (e.g. prodromal stage). Given the high NPV of HV (91.67%), it may be useful in ruling out AD among CU subjects. For CU subjects with a “positive” HV but a “negative” AD-RAI, confirmatory diagnostic test (e.g. PET, CSF analyses) can be further arranged. Hence, to detect preclinical AD, we may need to take into account of both HV and AD-RAI.

**Figure 2:**
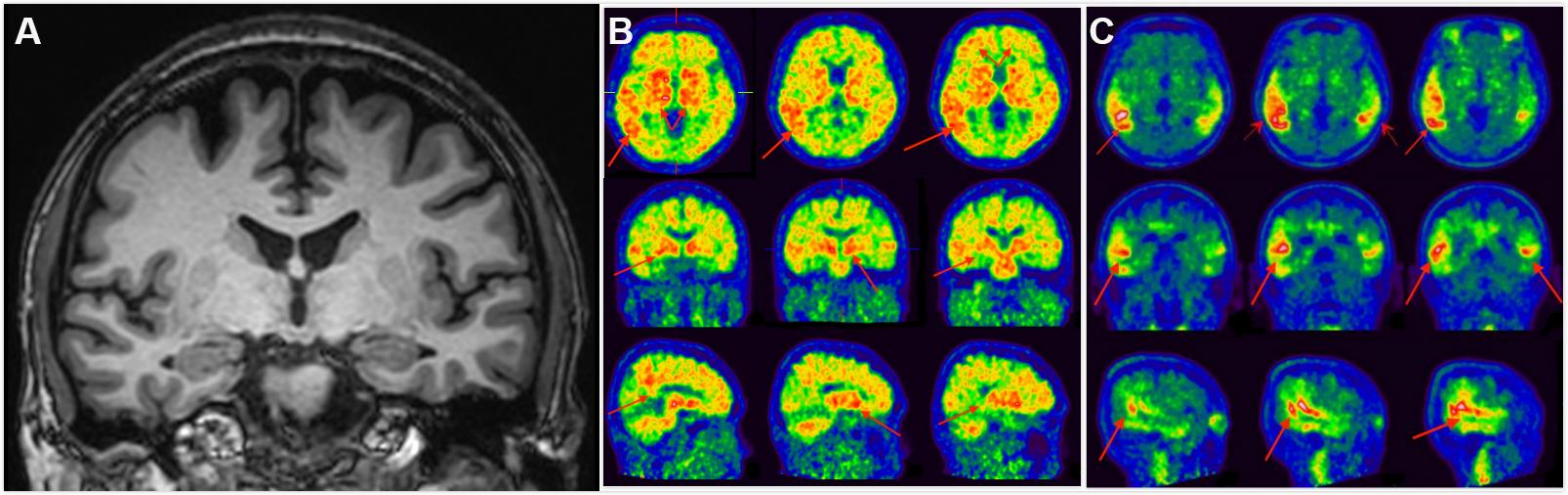
**Clinical utility of AD-RAI in MCI subjects.** A 50-year-old man with 16-year education had complaints of impaired short-term memory for 4 years. Z-score in Trial 4 of HKLLT was -2.97 SD (≤ -1 SD, i.e. MCI). The average visual MTA rating score was 0 suggestive of non-AD (**A**). HV measures also suggested non-AD, with a normal raw HV of 7.25mL (> 6.07mL) and HF of 0.48% (> 0.41%). However, AD-RAI was 0.68 (> 0.5) suggestive of AD. Subsequent PIB PET (**B**) and T807 PET (**C**) confirmed PIB and T807 retention, respectively (red arrows). The final diagnosis of this subject was prodromal AD. Abbreviations: AD-RAI=Alzheimer’s disease resemblance atrophy index; MCI=mild cognitive impairment; MTA=medial temporal lobe atrophy; HKLLT=Hong Kong List Learning Test; SD=standard deviation; MRI=magnetic resonance imaging; HV=hippocampal volume; HF=hippocampus fraction; PET=positron emission tomography; SVD=small vessel disease.

In this study, sensitivity of visual MTA rating in detecting AD at an early stage was low, which might partly be explained by the fact that the current visual grading has a floor effect (Figure 2). However, devising a finer visual scale may be challenging as detecting small volumetric change by human vision may not be possible and is also not reliable. The intra-rater reliability of this study obtained from an experienced neuroradiologist was 0.74 (weighted Kappa), which was compatible with that obtained from study among experienced neuroradiologist [25]. However, among general radiologists, the intra-rater reliability could be as low as 0.38 [25]. As a whole, if a finer visual scale is used, the reliability will likely be even lower. Note that the current machined-based automated tool had a test/re-test precision of 100%.

Among subjects having A+ with or without T+, the imaging measures had poorer performance when compared to that among subjects having both A+T+. This is expected because brain atrophy is likely absent or negligible when only beta-amyloid is present. Therefore, assessing brain atrophy using MRI is unlikely to be able to identify the earliest stage of the Alzheimer’s continuum, i.e. A+T-.

A strength of our study was that all our subjects received comprehensive clinical and imaging assessment, including amyloid and tau PET or CSF concentrations of Aβ_1–42_ and p-tau, hence allowing accurate classification on the cognitive, amyloid, and tau status of each individual. Another strength was that our study included participants from two separate cohorts involving different ethnicities. Majority of the participants were Caucasians in the ADNI cohort, while in CU-SEEDS, all were Chinese. Note that the performance of AD-RAI and HV was similar between these 2 cohorts, thus enhancing the generalizability of our findings. Our study has several limitations. Despite we had recruited more than a hundred CU and MCI subjects with clearly defined amyloid and tau status, our sample size was relatively small. In particular, among the CU subjects, only 15 were A+T+. Note however that a previous study showed that a sample size of 15 converters, who converted from CU to AD dementia (i.e. presumably A+T+) and 50 non-converters (presumably non-AD) were able to show a statistically significant difference in the volume of multiple brain regions. [11] Hence, our current sample size should be adequate to investigate the differentiating ability of AD-RAI and other HV measures. Yet, a larger study is needed to further validate the performance of AD-RAI and other HV measures in the detection of early AD. Another limitation was that the current threshold (0.5) of AD-RAI was generated based on subjects’ conversion status. Although the performance using the current threshold of 0.5 was good, we could not assume that those who converted to MCI or dementia were all A+T+, as other non-AD pathological process (e.g. cerebral small vessel disease) could also drive the conversion. Ideally, the optimal threshold of preclinical or prodromal AD will need to be derived from a larger cohort of CU and MCI subjects with clearly defined amyloid and tau status. Moreover, we used ^18^F-T807 PET for detection of tau pathology, off-target ^18^F-T807 bindings unrelated to tau in the basal ganglia [26, 27] or in some tau-negative conditions [28, 29] were reported. Note that in our study, we did not label subjects with ^18^F-T807 uptake at basal ganglia as T+. Ideally, the performance of automatic volumetric segmentation tool needs to be validated against brain pathology.

In conclusion, we validated an MRI-based machine learning derived AD-RAI at the cutoff of ≥ 0.5 in the detection of early AD, in particular at the prodromal stage. Given the validity, reliability, and ease of use, AD-RAI may provide additional information in guiding physicians or researchers of selecting who should receive further confirmatory investigations for the diagnosis of early AD as defined by the presence of A+ and T+, in particular among subjects presenting with MCI.

## MATERIALS AND METHODS

### Participants

Half of the participants of this study was recruited from an on-going CU-SEEDS (The Chinese University of Hong Kong - Screening for Early AlzhEimer’s DiseaSe) study, which aimed to validate biomarkers (e.g. retinal imaging, brain MRI, plasma) for detection of AD. The study aimed to initially recruit 100 subjects (40 CU, 40 MCI, 20 mild dementia) from the community and Cognitive Disorder Clinic of the Prince of Wales Hospital, Hong Kong SAR. Inclusion criteria were (1) Chinese ethnicity; (2) age between 50 to 80-year-old; and (3) a primary language of Cantonese. Exclusion criteria were (1) known diagnosis of non-AD dementia; (2) known history of stroke, parkinsonism, major psychiatric disease, or any significant neurological diseases (e.g. brain tumor); and/or (3) contraindication for MRI/PET. An experienced dementia specialist (L.W.C.A.) examined all potential subjects for eligibility of this study.

The other half of MCI and CU participants were recruited from ADNI cohort, excluding subjects from ADNI-2 who were used as the training cohort in our previous derivation study. Details on the ADNI cohort could be found online at: http://adni.loni.usc.edu.

### Syndromal staging of cognitive continuum of the participants

In CU-SEEDS, CU and MCI were defined according to the 2018 NIA-AA research framework [1]. We used the Chinese Abbreviated Memory Inventory (CAMI) to define the presence of memory complaints [30]. Subjects having one or more “Yes” to the 5 questions in CAMI were classified as having subjective memory complaints. We performed Hong Kong List Learning Test (HKLLT) [31] and the Hong Kong version of Montreal Cognitive Assessment (HK-MoCA) [32] for all subjects. We defined MCI as the presence of subjective memory complaints that represented a decline from baseline, objective memory impairment as defined by a z-score adjusted by age in Trial 4 (i.e. 10 min-delayed recall) of HKLLT of ≤ -1 standard deviation (SD) [33], and the cognitive impairment that has no major impact in daily function as defined by clinical dementia rating scale (CDR) of ≤ 0.5. We defined CU as having a z-score adjusted by age in Trial 4 of HKLLT > -1SD and a CDR of 0. Apart from MCI and CU subjects, we also recruited 10 dementia subjects for the purpose of validating our PET protocols. These 10 dementia subjects presented with AD-like dementia syndrome (i.e. episodic memory decline as the initial presentation, slowly progressive overtime, no atypical features such as motor deficits or parkinsonism) and had CDR of 1. They were diagnosed by an experienced dementia specialist (L.W.C.A.) All participants provided written informed consent and this study was approved by the local ethics committee.

In ADNI, CU subjects were defined as having Mini Mental State Examination (MMSE) scores between 24-30 (inclusive) and a CDR of 0 without depression, MCI and dementia. MCI subjects were defined as the presence of subjective memory complaints that represented a decline from baseline, having MMSE scores between 24-30 (inclusive) and a CDR of 0.5, and having objective memory loss measured by education-adjusted scores on a delayed logical memory score (9–11 for those with 16 or more years of education, 5–9 for 8–15 years of education, or 3–6 for 0–7 years of education, where possible scores range from 0 to 25), with absence of significant enough levels of impairments in other cognitive domains so that criteria for dementia are not met, largely preserved activities of daily living, and an absence of dementia. Details of inclusion and exclusion criteria could be found online at: http://adni.loni.usc.edu.

### MRI

MRI in CU-SEEDS cohort was performed at Prince of Wales Hospital using a 3.0 Tesla scanner (Achieva TX; Philips Medical Systems, Best, Netherlands). The scanning protocol included a 3D T1-weighted MPRAGE sequence acquired at a resolution of 1.1mmx1.1mmx1.2mm which was used for visual assessment and volumetric analysis, as well as standard T2-weighted and FLAIR sequences.

MRI in ADNI cohort were collected from http://adni.loni.usc.edu for further analyses. Imaging analyzed in our study was performed at 3.0 Tesla scanners including a 3D T1-weighted sequence which was used for visual rating and post-processing analysis, as well as T2-weighted and FLAIR sequences. Details could be referred to the website above.

### PET in CU-SEEDS cohort

We performed ^11^C- PIB and ^18^F-T807 PET/CT to quantify beta-amyloid and tau deposition, respectively at the Department of Nuclear Medicine and PET of Hong Kong Sanatorium and Hospital, Hong Kong SAR. All subjects received ^11^C-PIB intravenously and were scanned at 35 min post injection. Within one week, they underwent ^18^F-T807 PET/CT at 85 min post IV injection. ^11^C-PIB and ^18^F-T807 uptake were quantified by the “global cortical to cerebellum Standard Uptake Value ratio (SUVR)”. The calculation of SUVR included 13 target regions of interest contoured automatically: frontal gyrus, gyrus rectus, lateral temporal lobe, medial temporal lobe, posterior cingulate gyrus, precuneus, putamen, thalamus, superior parietal lobe, occipital lobe, head of the caudate, cerebellar vermis and brainstem.

We defined A+ if (1) increased ^11^C-PIB uptake was visually observed in regions known to have beta-amyloid deposits in the early stage of AD, i.e. posterior cingulate and/or precuneus with or without involvement of other brain regions (e.g. frontal lobes) [34] and/or (2) global retention ≥1.42 [35]. We defined T+ if (1) increased ^18^F-T807 uptake was visually observed in regions known to have tau deposits in the early stage of AD, i.e. medial temporal lobe, with or without involvement of other brain regions (e.g. rest of the temporal lobe, parietal lobe) [34, 36, 37] and/or (2) SUVR ≥1.14 [38]. CU and MCI subjects who had A+T+ based on PET findings were defined as having preclinical and prodromal AD, respectively [1]. All PET imaging data was interpreted by an experienced nuclear medicine specialist (E.Y.L.L.) who was blinded to subjects’ cognitive and structural imaging data.

### CSF biomarkers in ADNI cohort

CSF concentrations of Aβ_1–42_ and p-tau at baseline were obtained from http://adni.loni.usc.edu. We defined A+ if the concentration of Aβ_1–42_ was equal to or less than 192pg/ml [39]. We also defined T+ if the concentration of p-tau was equal to or above 23pg/ml [39]. CU and MCI subjects harboring A+T+ based on CSF findings were defined as have preclinical and prodromal AD, respectively [1].

### Visual ratings of MTA

An experienced neuroradiologist (J.A.) rated MTA using Scheltens’s scale [40] in both CU-SEEDS cohort and ADNI cohort. 10 individuals were randomly selected and rated again by the same neuroradiologist to obtain intra-rater reliability. We took the average of the left and right MTA scores as the final MTA score. We used the cutoff of ≥ 1 to define prodromal [41] and preclinical AD.

### MRI post-processing

All the MRIs from CU-SEEDS and ADNI were processed automatically using AccuBrain^®^ IV 1.1 (BrainNow Medical Technology Company Ltd.) that performs brain structure and tissue segmentation and quantification using 3D T1-weighted MR image [42]. This automatic post-processing method takes 20 minutes to generate AD-RAI and other quantitative measures. We used the summation of the volume of both sides in milliliter (mL) as the final raw HV. Accubrain^®^ also generated the hippocampal fraction (HF) (bilateral absolute HV/intracranial volume). AccuBrain^®^ also generated AD-RAI to indicate the similarity in atrophy pattern between the subject’s brain and those with AD with dementia (ranging from 0 to 1.0). Overall, AD-RAI is based on a machine learning method and it does not need extraction of radiomic features. Based on an in-house training database with the brain volumetric data of both normal subjects and AD dementia patients, AccuBrain® computes and selects the most relevant brain regional volumetry and projects the multi-dimensional brain regional volumetry features into a single atrophy index (i.e. AD-RAI) for the individual to be tested. The in-house training database contains brain MRI scans of 400 subjects, with 45% AD dementia patients and 55% CU subjects. Regarding the inclusion criteria of the in-house training database, for the AD group they were: (1) diagnosis of AD according to the International Classification of Diseases, 10th Revision (ICD-10), (2) CDR ≥ 1, (3) able to perform the neuropsychological test and tolerate the MRI scanning. The inclusion criteria for the CU group were: (1) normal in general physical status, (2) a CDR of 0 and (3) no memory complaints.

We investigated the performance of AD-RAI in detecting subjects with A+T+ using an index of ≥ 0.5, as obtained from the derivation study that was found to be the optimal cutoff in differentiating between “converters” and “stable” using ADNI-2 database [19]. Note that in our derivation study, we did not obtain the optimal cutoffs of HV and HF in differentiating between “converters” and “stable”. In order to compare AD-RAI with conventional imaging measures (i.e. HV and HF) in detecting A+T+ subjects in the present validation study, we further generated receiving operating curve (ROC) among all subjects with mild or no cognitive impairment (i.e. MCI and CU subjects) and among MCI and CU subgroups for the differentiation between “converters” and “stable” subjects. The derived optimal cutoffs were as follows: all subjects (i.e. MCI and CU) - HV: 6.44mL, HF: 0.42%; MCI subjects - HV: 6.07mL, HF: 0.41%; and CU subjects - HV: 6.64mL, HF: 0.44%. The performance metrics (sensitivity, specificity, positive predictive values, negative predictive values, accuracy) using the optimal cutoffs of AD-RAI, HV, and HF in differentiating converters and stable subjects from ADNI subjects can be found in Supplementary Table 1A–1C. MRI of the 10 individuals who were randomly selected for evaluation of intra-rater reliability for visual MTA rating were processed again by AccuBrain^®^ to test/re-test precision of the tool in generating HV, HF, and AD-RAI.

### Statistical analyses

Continuous variables were presented as means (SD), whilst categorical variables were presented as numbers (percentage). We compared the demographic characteristics of the MCI and CU subjects using independent-samples t-test for group comparisons. Intra-rater reliability was assessed with the weighted Cohen’s kappa test [43]. Sensitivity and specificity with 95% confidence intervals (CI), positive and negative prediction values (PPV, NPV), and accuracy were employed to evaluate the performance of four different imaging measures (i.e. AD-RAI, HV, HF, visual MTA) in the identification of A+T+ subjects among all subjects with MCI and CU (n=128), MCI subjects (n=50), and CU subjects (n=78). The metrics of various imaging measures in CU-SEEDS and ADNI cohorts were also calculated respectively. We also explored the metrics of various imaging measures in the detection of A+ with or without T+ (i.e. Alzheimer’s continuum). Statistical analyses were performed using SPSS version 25.0 for IOS.

## Supplementary Material

Supplementary Tables
