# Supplementary material for: MRI-based Alzheimer’s disease-resemblance atrophy index in the detection of preclinical and prodromal Alzheimer’s disease
Source: Aging (Albany NY). 2021 May 25;13(10):13496–514. doi: 10.18632/aging.203082 (PMC8202853; doi:10.18632/aging.203082)
Supplement: Supplementary Tables [file aging-13-203082-s001.pdf]

SUPPLEMENTARY TABLES

Supplementary Table 1A. Performance metrics of derived AD-RAI, HV and HF in differentiating converters and stable subjects from ADNI-2 database among all subjects (n=158).

Measures	Sensitivity (95% CI)	Specificity (95% CI)	Positive predictive value	Negative predictive value	Accuracy
AD-RAI (≥ 0.5)	0.64 (0.50-0.76)	0.79 (0.69-0.86)	63.79%	79.00%	73.42%
HV (≤ 6.44mL)	0.67 (0.53-0.79)	0.62 (0.52-0.71)	50.65%	76.54%	63.92%
HF (≤ 0.42%)	0.64 (0.50-0.76)	0.56 (0.46-0.66)	45.68%	72.73%	58.86%

AD-RAI=Alzheimer's disease resemblance atrophy index; HV=hippocampal volume; HF=hippocampal fraction; ADNI= Alzheimer's Disease Neuroimaging Initiatives; CI=confidence interval.

Supplementary Table 1B. Performance metrics of derived AD-RAI, HV and HF in differentiating converters and stable subjects from ADNI-2 database among MCI subjects (n=85).

Measures	Sensitivity (95% CI)	Specificity (95% CI)	Positive predictive value	Negative predictive value	Accuracy
AD-RAI (≥ 0.5)	0.80 (0.63-0.91)	0.60 (0.45-0.73)	58.33%	81.08%	68.24%
HV (≤ 6.07mL)	0.66 (0.48-0.80)	0.62 (0.47-0.75)	54.76%	72.09%	63.53%
HF (≤ 0.41%)	0.63 (0.45-0.78)	0.54 (0.39-0.68)	48.89%	67.50%	57.65%

AD-RAI=Alzheimer's disease resemblance atrophy index; HV=hippocampal volume; HF=hippocampal fraction; ADNI= Alzheimer's Disease Neuroimaging Initiatives; MCI=mild cognitive impairment; CI=confidence interval.

Supplementary Table 1C. Performance metrics of derived AD-RAI, HV and HF in differentiating converters and stable subjects from ADNI-2 database among NC subjects (n=73).

Measures	Sensitivity (95% CI)	Specificity (95% CI)	Positive predictive value	Negative predictive value	Accuracy
AD-RAI (≥ 0.5)	0.39 (0.20-0.61)	0.98 (0.88-1.00)	90.00%	77.78%	79.45%
HV (≤ 6.64mL)	0.70 (0.47-0.86)	0.62 (0.47-0.75)	45.71%	81.58%	64.38%
HF (≤ 0.44%)	0.65 (0.43-0.83)	0.58 (0.43-0.72)	41.67%	78.38%	60.27%

AD-RAI=Alzheimer's disease resemblance atrophy index; HV=hippocampal volume; HF=hippocampal fraction; ADNI= Alzheimer's Disease Neuroimaging Initiatives; NC=normal control; CI=confidence interval.

Supplementary Table 2. Performance metrics of AD-RAI, HV, HF and MTA among dementia subjects in CU-SEEDS cohort (n=10).

Measures	Sensitivity (95% CI)	Specificity (95% CI)	Positive predictive value	Negative predictive value	Accuracy
AD-RAI (≥0.5)	0.90 (0.54-0.99)	NA	100.00%	0	90.00%
HV (≤ 5.71mL*)	0.80 (0.44-0.96)	NA	100.00%	0	80.00%
HF (≤ 0.38%*)	0.50 (0.20-0.80)	NA	100.00%	0	50.00%
MTA (Adjusted by age**)	0.80 (0.44-0.96)	NA	100.00%	0	80.00%

AD-RAI=Alzheimer's disease resemblance atrophy index; HV=hippocampal volume; HF=hippocampal fraction; MTA=medial temporal lobe atrophy; CU-SEEDS= The Chinese University of Hong Kong - Screening for Early Alzheimer's Disease; CI=confidence interval. *The cutoff of HV (≤ 5.71mL) and HF (≤ 0.38%) used here were also derived from previous study [1]. **The cut-off scores of MTA: under 75 years ≥ 1.5 and ≥ 75 years ≥ 2 [2].

Supplementary Table 3A. Performance metrics of AD-RAI and MTA among MCI and CU subjects harboring A+ (n=128).

Measures	Sensitivity (95% CI)	Specificity (95% CI)	Positive predictive value	Negative predictive value	Accuracy
AD-RAI (≥0.5)	0.65 (0.49-0.77)	0.94 (0.85-0.98)	86.11%	81.52%	82.81%
HV (≤ 6.44mL)	0.65 (0.49-0.77)	0.78 (0.67-0.86)	63.27%	78.48%	72.66%
HF (≤ 0.42%)	0.46 (0.32-0.61)	0.94 (0.85-0.98)	81.48%	74.26%	75.78%
MTA (≥ 1)	0.48 (0.34-0.63)	0.90 (0.81-0.95)	74.19%	74.23%	74.22%

AD-RAI=Alzheimer's disease resemblance atrophy index; HV=hippocampal volume; HF=hippocampal fraction; MTA=medial temporal lobe atrophy; CI=confidence interval.

Supplementary Table 3B. Performance metrics of AD-RAI, HF, HV and MTA among MCI subjects harboring A+ (n=50).

Measures	Sensitivity (95% CI)	Specificity (95% CI)	Positive predictive value	Negative predictive value	Accuracy
AD-RAI (≥ 0.5)	0.86 (0.66-0.95)	0.86 (0.64-0.96)	88.89%	82.61%	86.00%
HV (≤ 6.07mL)	0.68 (0.48-0.83)	0.95 (0.75-1.00)	95.00%	70.00%	80.00%
HF (≤ 0.41%)	0.54 (0.34-0.72)	0.95 (0.75-1.00)	93.75%	61.76%	72.00%
MTA (≥ 1)	0.64 (0.44-0.81)	0.86 (0.64-0.96)	85.71%	65.52%	74.00%

AD-RAI=Alzheimer's disease resemblance atrophy index; HV=hippocampal volume; HF=hippocampal fraction; MTA=medial temporal lobe atrophy; MCI=mild cognitive impairment; CI=confidence interval.

Supplementary Table 3C. Performance metrics of AD-RAI, HF, HV and MTA among CU subjects harboring A+ (n=78).

Measures	Sensitivity (95% CI)	Specificity (95% CI)	Positive predictive value	Negative predictive value	Accuracy
AD-RAI (≥ 0.5)	0.35 (0.16-0.59)	0.97 (0.87-0.99)	77.78%	81.16%	80.77%
HV (≤ 6.64mL)	0.70 (0.46-0.87)	0.91 (0.80-0.97)	73.68%	89.83%	85.90%
HF (≤ 0.44%)	0.35 (0.16-0.59)	0.97 (0.87-0.99)	77.78%	81.16%	80.77%
MTA (≥ 1)	0.25 (0.10-0.49)	0.91 (0.80-0.97)	50.00%	77.94%	74.36%

AD-RAI=Alzheimer's disease resemblance atrophy index; HV=hippocampal volume; HF=hippocampal fraction; MTA=medial temporal lobe atrophy; CU=cognitively unimpaired; CI=confidence interval.

SUPPLEMENTARY REFERENCES

1. Abrigo J, Shi L, Luo Y, Chen Q, Chu WCW, Mok VCT; Alzheimer's Disease Neuroimaging Initiative. Standardization of hippocampus volumetry using automated brain structure volumetry tool for an initial Alzheimer's disease imaging biomarker. Acta Radiol. 2019; 60:769–76. https://doi.org/10.1177/0284185118795327 PMID:30185071

2. Pereira JB, Cavallin L, Spulber G, Aguilar C, Mecocci P, Vellas B, Tsolaki M, Kłoszewska I, Soininen H, Spenger C, Aarsland D, Lovestone S, Simmons A, Wahlund LO, Westman E; AddNeuroMed consortium and for the Alzheimer's Disease Neuroimaging Initiative. Influence of age, disease onset and ApoE4 on visual medial temporal lobe atrophy cut-offs. J Intern Med. 2014; 275:317–30. https://doi.org/10.1111/joim.12148 PMID:24118559
